# Economic and Social Influences on Later-Life Well-Being: New Evidence From the NLSY79

**DOI:** 10.1093/geroni/igaa057.1951

**Published:** 2020-12-16

**Authors:** Deborah Carr, Stephanie Burge

## Abstract

The National Longitudinal Survey of Youth 1979 (NLSY79) provides unprecedented opportunities for understanding how work, family, and socioeconomic characteristics over a 40-year period shape the well-being of older adults. The large sample enables explorations of race, gender, and socioeconomic differences in these processes. The five papers in this symposium exploit the rich life course data of NLSY79 to understand two key outcomes: health, and work/economic arrangements as adults approach their retirement years. Harrati and Heburn document the impacts of unemployment trajectories on physical and mental health, highlighting gender differences in these processes. Wolfe investigates the long-term health consequences of significant economic setbacks over the life course (shocks), taking into account those risk factors (selection) that render one vulnerable to such shocks. Jang and Tang document the negative impacts of informal caregiving on later-life physical health, yet their subgroup analyses detect positive impacts for African-Americans. Aughinbaugh delineates women’s time spent in caregiving over the life course, and discusses the implications for later-life economic well-being of both early life childcare and later-life coresidential caregiving. Walsemann, Fisk and Ailshire examine the role that parents and grandparents play in paying for their offspring’s college education, with careful attention to gender and socioeconomic differences in these patterns. Taken together, these papers underscore the importance of adopting an intersectional approach to understanding later-life well-being, and reveal the complex ways that paid work, unpaid work (caregiving), and inter-transfers (via tuition payment) bear on the health and financial stability of adults transitioning into their retirement years.

